# Late onset deficits in synaptic plasticity in the valproic acid rat model of autism

**DOI:** 10.3389/fncel.2014.00023

**Published:** 2014-01-31

**Authors:** Henry G. S. Martin, Olivier J. Manzoni

**Affiliations:** ^1^INSERM U901 Pathophysiology of Synaptic Plasticity GroupMarseille, France; ^2^Institut de Neurobiologie de la Méditerranée (INMED)Marseille, France; ^3^Université de Aix-MarseilleMarseille, France

**Keywords:** valproic acid, prefrontal cortex, synaptic plasticity, autism, age, NMDA receptor

## Abstract

Valproic acid (VPA) is a frequently used drug in the treatment of epilepsy, bipolar disorders and migraines; however it is also a potent teratogen. Prenatal exposure increases the risk of childhood malformations and can result in cognitive deficits. In rodents *in utero* exposure to VPA also causes neurodevelopmental abnormalities and is an important model of autism. In early postnatal life VPA exposed rat pups show changes in medial prefrontal cortex (mPFC) physiology and synaptic connectivity. Specifically, principal neurons show decreased excitability but increased local connectivity, coupled with an increase in long-term potentiation (LTP) due to an up-regulation of NMDA receptor (NMDAR) expression. However recent evidence suggests compensatory homeostatic mechanisms lead to normalization of synaptic NMDARs during later postnatal development. Here we have extended study of mPFC synaptic physiology into adulthood to better understand the longitudinal consequences of early developmental abnormalities in VPA exposed rats. Surprisingly in contrast to early postnatal life and adolescence, we find that adult VPA exposed rats show reduced synaptic function. Both NMDAR mediated currents and LTP are lower in adult VPA rats, although spontaneous activity and endocannabinoid dependent long-term depression are normal. We conclude that rather than correcting, synaptic abnormalities persist into adulthood in VPA exposed rats, although a quite different synaptic phenotype is present. This switch from hyper to hypo function in mPFC may be linked to some of the neurodevelopmental defects found in prenatal VPA exposure and autism spectrum disorders in general.

## Introduction

Due to its anti-convulsant action and mood stabilizing properties valproic acid (VPA) is a common treatment for bipolar disorder and childhood epilepsy (McElroy et al., 1989). These stabilizing properties have been attributed to the action of VPA on GABA transaminobutyrate and sodium ion channels, however more recently VPA has been described as a histone deacetylase inhibitor (Göttlicher et al., 2001) leading to renewed interest in VPA for the treatment of a wide range of psychiatric and non-psychiatric diseases (Chateauvieux et al., 2010). Unfortunately VPA is also a potent teratogen and prenatal exposure increases the risk of congenital malformations and neural tube defects (Meador et al., 2008). Specifically VPA exposure *in utero* results in neurodevelopmental delays apparent in poor verbal performance and cognitive impairments (Nadebaum et al., 2011; Meador et al., 2013). Furthermore prenatal VPA exposure is associated with a seven-fold increased risk of developing autism spectrum disorders and is a significant prenatal hazard (Rasalam et al., 2005; Bromley et al., 2008; Christensen et al., 2013).

*In utero* injection of VPA during neural tube closure in rats and mice results in progeny that model some of the neurodevelopmental changes found in humans. Most prominent is an increase in autistic like behaviors in VPA exposed rodents; notably increased repetitive behaviors, reduced social interaction and hypersensitivity (Schneider and Przewłocki, 2005; Dufour-Rainfray et al., 2010; Gandal et al., 2010; Kim et al., 2011; Mehta et al., 2011). These behaviors have led to the proposal that *in utero* exposure to VPA may represent a useful rodent model of autism and is the basis of the “intense world theory” of autism (Markram et al., 2007).

Changes in local and distant connectivity in the brain have been proposed as a possible cause of autistic behavior (Geschwind and Levitt, 2007). Using the *in utero* VPA exposure model, local changes in principal neuron connectivity and excitability have been found in the rat medial prefrontal cortex (mPFC; Rinaldi et al., 2008a,b). This is perhaps particularly pertinent since the mPFC is linked to autistic behaviors and mPFC abnormalities are found in many neuropsychiatric disorders (Goto et al., 2010). The mPFC synaptic physiology in the VPA exposed rat pups appears to be tuned to a hyper-connected, hyper-excitable state, notable for an increase in NMDA receptor (NMDAR) synaptic expression and an enhancement of long-term potentiation (LTP; Rinaldi et al., 2007, 2008b; Kim et al., 2013). However, recent recordings from older adolescent VPA exposed rats (P30 days) have suggested a normalization of both synaptic physiology and neuronal excitability to naïve levels as pups develop (Walcott et al., 2011). In common with autism spectrum disorders, behavioral deficits are present throughout life in the rat VPA model (Roullet et al., 2013), however a description of adult synaptic physiology is lacking making it unclear if the synaptic compensatory mechanisms found at P30 extend into adulthood.

In this study we have examined synaptic physiology in the mPFC of the prenatally VPA exposed rat from adolescence into adulthood. Surprisingly we find a reversal of the enhanced synaptic NMDAR expression phenotype found in VPA rat pups; such that adult VPA exposed neurons show a deficit in NMDAR mediated currents. Furthermore these adult neurons show a loss of LTP compared to controls, but unaltered long-term depression (LTD).

## Materials and methods

### Animals

All animals were group housed with 12 h light/dark cycles in compliance with the European Communities Council Directive (86/609/EEC). Time-mated female Wistar rats received a single intra-peritoneal dose of 600 mg/kg VPA (Sigma; prepared as 300 mg/ml saline solution) at gestational day E12 (Schneider and Przewłocki, 2005). Control dams received a single similar volume injection of saline at the same gestational time-point. Adolescent rats were P48 ± 2 days (VPA: 3 males, 1 litter; Saline: 3 males, 1 litter); adult rats were P120 ± 10 days (VPA: 9 males, 2 litters; Saline: 7 males, 2 litters).

### Slice preparation and electrophysiology

After isoflurane anesthetization and decapitation, brains were sliced (300 μm) in the coronal plane in a sucrose-based solution (in mM: 87 NaCl, 75 sucrose, 25 glucose, 4 KCl, 2.1 MgCl_2_, 0.5 CaCl_2_, 18 NaHCO_3_ and 1.25 NaH_2_PO_4_). Slices were allowed to recover for 60 min at 32–35°C in artificial cerebrospinal fluid (aCSF; 126 NaCl, 2.5 KCl, 2.4 MgCl_2_, 1.2 CaCl_2_, 18 NaHCO_3_, 1.2 NaH_2_PO_4_ and 11 glucose; equilibrated with 95% O_2_/5% CO_2_) before transfer to the recording chamber.

**Figure 1:**
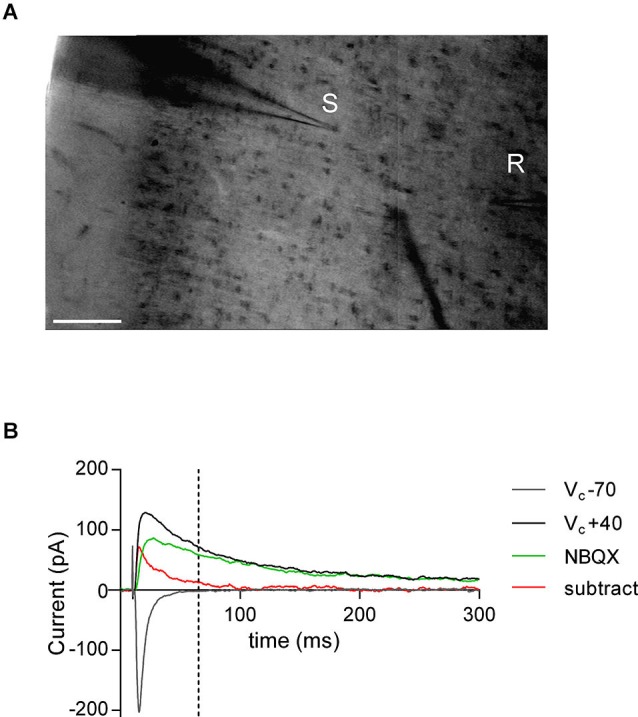
**AMPA—NMDA ratio experimental design. (A)** Bright-field snapshot of coronal slice detailing prelimbic mPFC division (*x*-axis medial—lateral; *y*-axis dorsal—ventral). Typical positioning of stimulation electrode (S) and patch-clamp recording electrode (R) are indicated (scale bar 100 μm). **(B)** Example traces from AMPA—NMDA ratio calculation. NMDAR mediated current was taken as V_C_ +40 mV value 50 ms after peak AMPAR mediated current measured at −70 mV (indicated by dashed line). NBQX insensitive current (green) and subtracted current (red) at V_C_ +40 mV show that AMPAR mediated currents are close to zero at NMDAR current measurement point.

Whole-cell patch-clamp and extra-cellular field recordings were made from layer V/VI pyramidal cells in coronal slices of prelimbic PFC (Lafourcade et al., 2007). For recording, slices were superfused (2 ml/min) with aCSF. All experiments were performed at 32–35°C. The recording aCSF contained picrotoxin (100 μM, Sigma) to block GABA_A_ receptors. To evoke synaptic currents, 150–200 μs stimuli were delivered at 0.1 Hz through an aCSF-filled glass electrode positioned dorsal-medial to the recording electrode in layer V (Figure 1A). Pyramidal neurons were visualized using an infrared microscope (BX-50, Olympus). Patch-clamp experiments were performed with electrodes filled with a cesium methane-sulfonate based solution (in mM; 143 CH_3_O_3_SCs, 10 NaCl, 1 MgCl_2_, 1 EGTA, 0.3 CaCl_2_, 2 Na^2+^-ATP, 0.3 Na^+^-GTP, 10 glucose buffered with 10 4-(2-hydroxyethyl)-1-piperazineethanesulfonic acid (HEPES), pH 7.3, osmolarity 290 mOsm). Prior to break-through into the cell, pipette capacitance was compensated and the reference potential of the amplifier was adjusted to zero. Junction potentials were not corrected. Electrode resistance was 3–5 MOhm. If access resistance was greater than 20 MOhm or changed by >20% during the period of recording, the experiment was rejected. During recording holding currents, series resistance and membrane time constant (τ) were monitored. Only monosynaptic excitatory post synaptic currents (EPSCs) were recorded with a latency of <5 ms. In extracellular field experiments, the recording pipette was filled with aCSF. The glutamatergic nature of the field excitatory postsynaptic potential (fEPSP) was confirmed at the end of the experiments using the ionotropic glutamate receptor antagonist 6,7-dinitroquinoxaline-2,3-dione (DNQX, 20 μM; National Institute of Mental Health Chemical Synthesis and Drug Supply Program (NIMH)).

Input-output profiles were recorded for all fEPSP recordings. For time course experiments the stimulation intensity was that necessary give a response 40–60% of the maximal. fEPSPs were recorded at 0.1 Hz. Using the same stimulation intensity as baseline, LTP was induced by a repeated (4 times, 10 s interval) theta burst stimulation (TBS; 4 100 Hz pulses repeated 5 times separated by 200 ms). LTD was likewise induced by a steady 10 Hz stimulation for 10 min.

### Data acquisition and analysis

Data was recorded on a MultiClamp700B (Axon Instruments), filtered at 2 kHz, digitized (10 kHz, DigiData 1440A, Axon Instrument), collected using Clampex 10.2 and analyzed using Clampfit 10.2 (all from Molecular Device, Sunnyvale, USA). Analysis of both area and amplitude of fEPSPs and EPSCs was performed.

The magnitude of LTP and LTD was calculated 25–30 min after tetanus as percentage of baseline responses. To determine the AMPA/NMDA ratio, the AMPAR component amplitude was measured from EPSC at −70 mV. The NMDAR component amplitude was determined 50 ms after the peak AMPAR-evoked EPSC at +40 mV, when the AMPAR component is over (Kasanetz and Manzoni, 2009). In a subset of experiments the AMPAR mediated current was inhibited with the selective antagonist 2,3-dihydroxy-6-nitro-7-sulfamoyl-benzo[f]quinoxaline-2,3-dione (NBQX, 10 μM; NIMH) to confirm the absence of an AMPAR contribution to the measured NMDAR component (Figure 1B). Spontaneous EPSCs were analyzed with Axograph X (Axograph). Statistical analysis of data was performed with GraphPad Prism (GraphPad Software Inc., La Jolla, CA) using tests indicated in the main text after Grubbs’ outlier subtraction (99% confidence). All values are given as mean ± standard error, *n* values represent individual animals and statistical significance was set at ^*^ *p* < 0.05 and ^**^ *p* < 0.01.

## Results

### Late onset deficits in synaptic currents are found in the medial prefrontal cortex (mPFC) of adult Valproic acid (VPA) exposed rats

Recently it has been reported that mPFC hyper-function found in juvenile rats exposed to the teratogen VPA is corrected in adolescent rats (Walcott et al., 2011). If such a strong compensatory mechanism exists during pup development, we asked if these modifications persist into adulthood. We focused on glutamatergic synapses of principal neurons in layer V/VI of the prelimbic region of the mPFC. These output neurons not only show a full range of synaptic plasticity throughout development into adulthood, but are also implicated in mPFC linked synaptopathologies (Lafourcade et al., 2011; Iafrati et al., 2013; Kasanetz et al., 2013).

**Figure 2:**
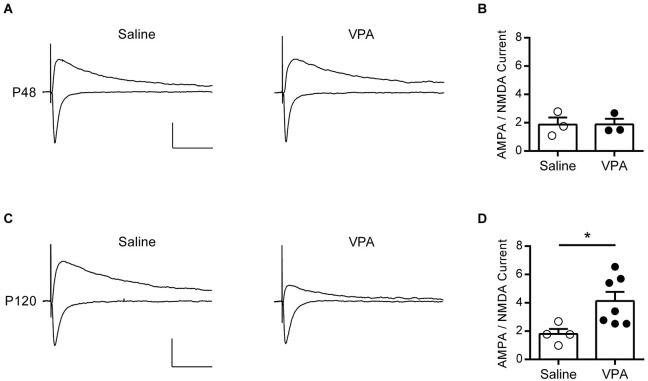
**AMPA—NMDA ratio is increased in adult but not adolescent VPA mPFC neurons. (A)** Example EPSCs recorded from visually identified pyramidal neurons in layer V/VI mPFC from adolescent rats (P45–P49). Negative traces represent inward evoked currents from neurons patch-clamped at −70 mV, positive traces represent outward evoked currents from neurons patch-clamped at +40 mV. Scale bar 50 ms, 100 pA. **(B)** Average AMPA/NMDA ratio from adolescent rats exposed to saline or VPA *in utero*. **(C)** Similar evoked example EPSC traces from visually identified deep layer pyramidal neurons from adult rats (P110–P130). **(D)** Average AMPA/NMDA ratio from adult rats exposed to saline or VPA *in utero*. Data points represent AMPA/NMDA ratio from individual animals. Data shown as mean ± s.e.m.; * *p* < 0.05.

We first verified, as reported by Walcott et al. (2011) that synaptic gain function is normalized in adolescent VPA exposed rats by recording evoked synaptic AMPAR and NMDAR currents and using the ratio of the two events as a measure of their modification. Neurons from adolescent rats exposed either to VPA or saline *in utero* reliably showed evoked EPSCs when voltage-clamped at both −70 mV (negative deflections) and +40 mV (positive defections, Figure 2A). In the mPFC, at −70 mV fast inward EPSCs are principally AMPAR mediated, whereas at +40 mV a mixed AMPAR and NMDAR outward current is detected. Thus we calculated an index for the ratio AMPAR to NMDAR mediated currents (AMPA-NMDA ratio) by dividing the maximal amplitude of the response at −70 mV (AMPA), by the +40 response amplitude at a predetermined time point after the fast AMPAR event had decayed to zero (NMDA). In agreement with Walcott et al. (2011) we find that this measure is broadly the same in both saline and VPA exposed neurons in adolescent rats (Figure 2B).

Given this compensatory rebalancing of synaptic function in adolescent rats, we asked if a similar effect is found in adult rats exposed to VPA *in utero*. At P120 strong EPSCs could be evoked from deep layer mPFC neurons clamped at −70 mV in both saline and VPA treated rats, however responses recorded at +40 mV were notably reduced in VPA neurons (Figure 2C). Repeating the same measure used in the adolescent rats, we calculated the AMPA-NMDA in our adult animals (Figure 2D). Surprisingly we found that in rats exposed to VPA *in utero* there was a significant increase in the AMPA-NMDA ratio compared to saline controls (*p* = 0.024; Mann-Whitney *U*-test). Notably saline treated adult rats had an AMPA-NMDA ratio similar to the value calculated for adolescents, whereas VPA treated rats had an elevated AMPA-NMDA ratio compared to both of these groups. Therefore in contrast to the reported reduction in AMPA-NMDA ratio found in mPFC of juvenile VPA exposed rats (Rinaldi et al., 2007, 2008a), we find an increase in this index in adulthood.

**Figure 3:**
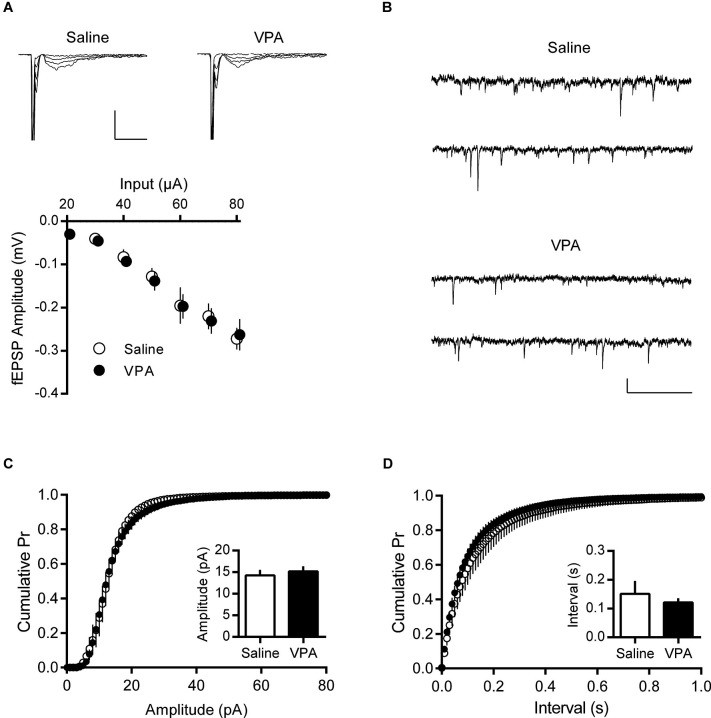
**Basal synaptic activity is normal in adult VPA neurons. (A)** Input—Output relationships derived from evoked fEPSPs in deep layer mPFC. Sample fEPSP traces from saline and VPA treated rats showing fEPSP change in response to 10 pA stimulation steps (top). Scale bar 10 ms, 0.5 mV. Average fEPSP amplitude from saline and VPA neurons (bottom). **(B)** Spontaneous EPSCs from pyramidal neurons patch-clamped at −70 mV. Scale bar 0.5 s, 10 pA. **(C)** Cumulative probability plot of individual spontaneous EPSCs from adult saline and VPA neurons. Average amplitude EPSC from individual animals (insert; Saline, *n* = 5; VPA, *n* = 7). **(D)** Cumulative probability plot of intervals between spontaneous EPSC events from saline and VPA neurons. Average recorded interval between spontaneous events from individual neurons (insert). Data shown as mean ± s.e.m.

Anecdotally the increase in AMPA-NMDA ratio found in adult VPA neurons appeared to be due to a change in NMDAR mediated currents; however a change in this index can be linked to a number of other synaptic parameters. Therefore we further characterized some of the basic properties of these synapses. Taking a systematic approach, we first measured field EPSPs (fEPSP) from layer V/VI neurons to build input-output profiles in saline and VPA neurons. fEPSPs evoked by electrical stimulation in the same layer showed a similar profile in response to increasing stimulation intensity (Figure 3A). Furthermore input-output curves from saline and VPA exposed neurons were nearly identical. Therefore these synapses do not show and gross changes in excitability.

To further characterize these synapses, we measured quantal events by recording spontaneous EPSCs (sEPSC). Both saline control and VPA neurons showed robust sEPSCs in adulthood (Figure 3B). We used the cumulative distribution of the amplitude of events to gauge any differences between the two groups (Figure 3C). However both the distribution and the mean amplitude of spontaneous events were broadly the same in VPA exposed and saline control neurons. At resting membrane potential (−70 mV) these events are principally AMPAR mediated, therefore as previously reported in adolescent animals (Walcott et al., 2011), VPA exposure does not appear to strongly effect AMPAR currents. Likewise, we compared the frequency of sEPSC by comparing the cumulative distribution of the interval between events (Figure 3D). Both saline and VPA exposed neurons had a similar distribution and average interval between events. Therefore both presynaptic release and postsynaptic AMPAR are similar in control and VPA exposed neurons in the mPFC.

### Adult Valproic acid (VPA) medial prefrontal cortex (mPFC) neurons have deficits in long-term potentiation (LTP), but not long-term depression (LTD)

**Figure 4:**
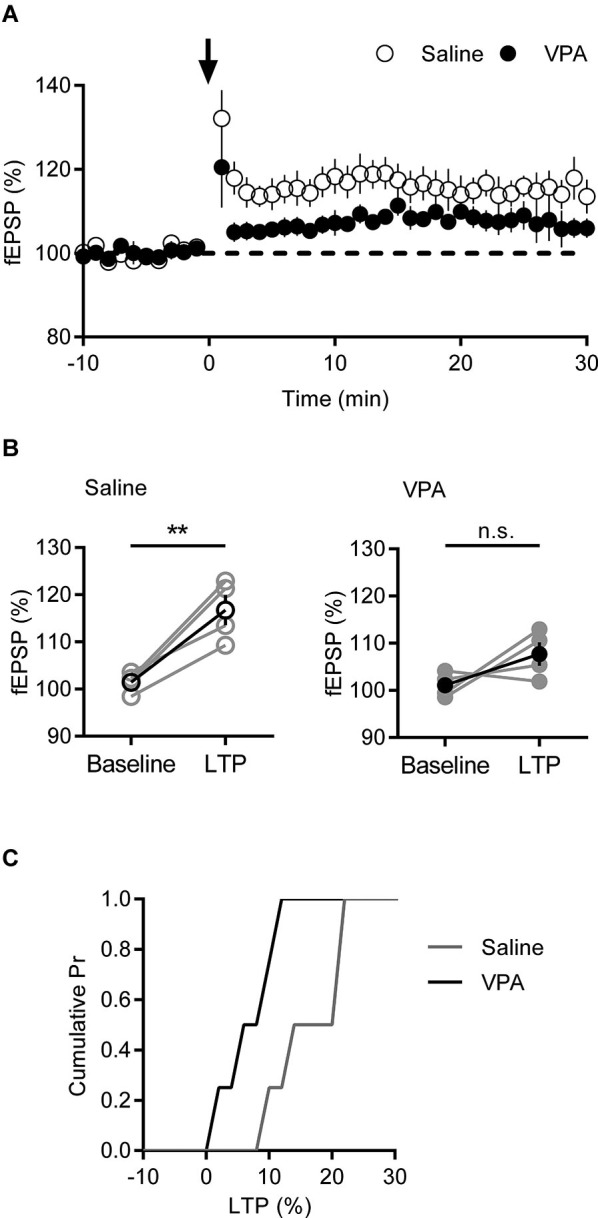
**Deficits in LTP are present in VPA exposed mPFC neurons. (A)** Time course of normalized fEPSP responses from layer V/VI adult rats treated with saline or VPA *in utero*. Theta burst LTP stimulation is indicated by arrow. **(B)** Change in normalized fEPSP pre- (Baseline) and post- (LTP; 25 min after TBS) theta burst stimulation LTP. Darker points represent the mean fEPSP. **(C)** Cumulative probability plot of percent LTP from individual experiments. Data shown as mean ± s.e.m.; ** *p* < 0.01.

In juvenile rats exposed to VPA, a decrease in AMPA-NMDA ratio was linked to increased LTP in the mPFC (Rinaldi et al., 2007). Since in adult VPA exposed rats we observe the opposite phenomenon, we asked if adult VPA rats might instead show reduced LTP. We recorded fEPSPs in layer V/VI and challenged slices with a short TBS LTP protocol; this induces a postsynaptic NMDAR dependent form of LTP (Iafrati et al., 2013). In saline controls a robust potentiation of fEPSPs was observed that was stable over the period of recording. In contrast in recordings from VPA exposed neurons, fEPSPs were smaller than saline controls (Figure 4A). Taking the fEPSP strength pre- and post-TBS we compared LTP in saline and VPA treated rats (Figure 4B). In control slices TBS induced a significant LTP (*p* = 0.012; Paired *t*-test), whereas in VPA slices there we did not detect a significant potentiation (*p* = 0.177; Paired *t*-test). Converting the post-TBS fEPSP to a percent LTP, we plotted the cumulative distribution of the percent LTP in individual experiments. The distribution of VPA exposed neurons is left shifted compared to saline controls, indicating a reduction in LTP (Figure 4C). Therefore in contrast to juvenile VPA exposed neurons in the mPFC, adult neurons show a reduction in NMDAR mediated LTP.

**Figure 5:**
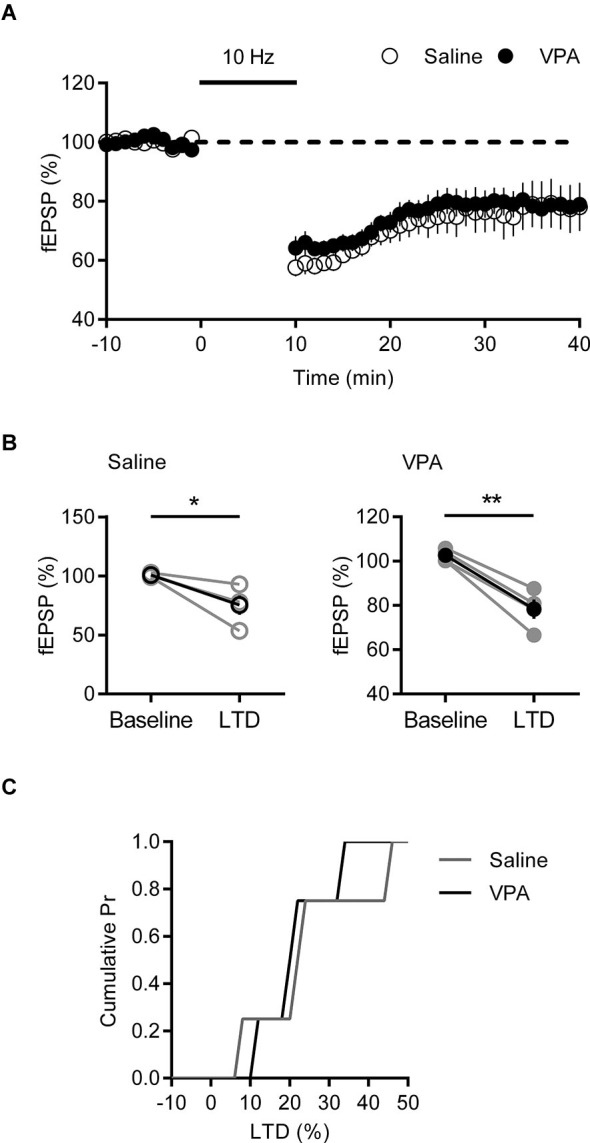
**LTD is normal in VPA exposed mPFC neurons. (A)** Time course of normalized fEPSP responses from layer V/VI adult rats treated with saline or VPA *in utero*. 10 Hz eCB-LTD stimulation is indicated by bar. **(B)** Change in normalized fEPSP pre- (Baseline) and post- (LTD; 25 min after 10 Hz stimulation) eCB-LTD. Darker points represent the mean fEPSP. **(C)** Cumulative probability plot of percent LTD from individual experiments. Data shown as mean ± s.e.m.; * *p* < 0.05, ** *p* < 0.01.

To confirm this reduction in LTP is a specific phenomenon linked to our observed changes in AMPA-NMDA ratio, we tested another form of activity dependent plasticity that is independent of NMDARs in the mPFC. Endocannabinoid-dependent long-term depression (eCB-LTD) induced by steady 10 Hz stimulation, engages an mGluR5 mediated mechanism that requires retrograde 2-AG signaling to presynaptic cannabinoid receptor type 1 (CB1) receptors (Lafourcade et al., 2007, 2011). Recording fEPSPs from mPFC deep layers, we induced eCB-LTD in saline and VPA *in utero* treated adult rats. Both groups showed an initial strong depression in fEPSP in response to 10 Hz stimulation that stabilized at approximately 80% of the baseline response (Figure 5A). Again we compared the response pre- and post-eCB-LTD (Figure 5B). In both saline controls and VPA exposed neurons we saw a significant depression of fEPSP (Saline: *p* = 0.042; VPA: *p* = 0.005; Paired *t*-test). Converting fEPSPs to percent LTD we compared the amount of depression in the two groups. Unlike LTP, the distribution and strength of LTD was similar between control and VPA neurons (Figure 5C). Therefore eCB-LTD is unaffected in adult rats exposed to VPA *in utero*.

## Discussion

In this study we have traced changes in mPFC synaptic physiology in the VPA rat from adolescence to adulthood. Juvenile rats exposed *in utero* to the teratogen VPA have a hyper-connected mPFC with enhanced NMDAR function (Rinaldi et al., 2007, 2008b). However recent evidence suggests that this phenotype is normalized as pups reach puberty (Walcott et al., 2011). Given the late maturation of the PFC, we wished to extend this synaptic description into adulthood to better understand the developmental consequences of VPA exposure. Surprisingly in contrast to VPA rat pups and adolescents, we found evidence of synaptic hypo-function during adulthood.

Compared to controls, VPA neurons in the mPFC show a reduced and delayed increase in AMPA-NMDA ratio over early postnatal development (Rinaldi et al., 2007; Walcott et al., 2011). However by the time rats reach early adolescence (P30) this parameter is similar in both VPA and saline controls. In concord we find that at puberty (P40–P50) that the AMPA-NMDA ratio is not significantly different between control and VPA rats in the mPFC. However, when we extended measurement of the AMPA-NMDA ratio into young adulthood (P110–P130) we found a significant increase of this index in VPA rats, but not controls.

The AMPA-NMDA ratio is a strong measure of synaptic state, particularly the relative number of AMPARs and NMDARs in the post-synapse (Watt et al., 2000), however other synaptic variables may also be involved. Therefore to better interpret the unexpected increase in AMPA-NMDA ratio in adult VPA rats, we measured quantal mPFC activity. Focusing on AMPAR mediated events we recorded spontaneous EPSCs in layer V/VI neurons. We found that neither the average amplitude nor distribution of spontaneous events was different in VPA and saline controls, indicating that synaptic AMPAR are unchanged in these rats. Likewise we failed to observe any change in frequency of spontaneous events. Superficially this is inconsistent with the local hyper-connectivity reported in the mPFC of juvenile VPA rats (Rinaldi et al., 2008b). However our measurements of spontaneous activity do not distinguish between local and distant connections, thus a relative reduction in distant connectivity could account for this inconsistency (Geschwind and Levitt, 2007; Rinaldi et al., 2008b).

The simplest interpretation of our AMPA-NMDA ratio in light of unchanged spontaneous activity is that NMDAR currents are reduced in adult rats exposed to VPA *in utero.* In the young VPA rat, Rinaldi et al. (2007) directly linked a decrease in AMPA-NMDA ratio to an increase in expression of GluN2a and GluN2b and a subsequent increase in LTP. Our increase in AMPA-NMDA ratio in the adult VPA rat was suggestive that the opposite phenomenon might occur in these older animals. Indeed, when we challenged deep layer mPFC neurons with TBS LTP protocol there was a measurable loss in potentiation with VPA exposure compared to saline controls. A similar NMDAR linked loss of LTP is found in a number of mouse genetic models of autism (Ebert and Greenberg, 2013; Jiang and Ehlers, 2013), suggesting the adult VPA rat shares similarities with these mice. We found no change in mGluR5 mediated eCB-LTD in these synapses, demonstrating that this deficit in LTP is not a general loss of synaptic gain function.

Fragile X syndrome (FXS) is a genetic disorder with a complex endophenotype that often includes autism linked behaviors (Cornish et al., 2008). Similar to our findings in VPA rats, FXS mice show a reduction in NMDAR expression in the mPFC and a loss of LTP (Zhao et al., 2005; Meredith et al., 2007; Krueger et al., 2011). Likewise in young FXS mice (2–3 weeks) mGluR5 mediated LTD is unaffected (Desai et al., 2006; Meredith et al., 2007), although in adult mice a deficit in coupling between mGluR5 activation and retrograde signaling appears to be responsible for a loss in eCB-LTD (Jung et al., 2012). Similar to the VPA rat, age and development linked deficits in synaptic physiology appear to be present in the FXS mouse, although differences in early postnatal life suggest these two models of autism are not directly comparable (Desai et al., 2006; Rinaldi et al., 2007).

The surprising finding in this study is that rats prenatally exposed to VPA pass from an enhanced LTP phenotype in early life to a LTP deficit phenotype in adulthood. Immediately before puberty and during adolescence NMDAR mPFC physiology appears similar to saline control suggesting that VPA neurons transition through a normal period between a hyper to hypo synaptic function. It has been proposed that homeostatic compensatory mechanisms may be responsible for the initial normalization of mPFC neuronal function in VPA rats (Walcott et al., 2011)*.* How these compensatory synaptic scaling mechanisms work is unclear, however our results suggest that the action of normalizing hyper function in early life may subsequently lead to the loss of LTP in adulthood. Such a rebound effect may be due to the engagement of feedback loops that act over extended periods of development. It is also noteworthy that in our recordings we average across layer V/VI neuronal responses. This is significant since layer V principal neurons appear to belong to one of two subtypes with differing intrinsic properties and output projects (Dembrow et al., 2010; Lee et al., 2014). Regardless our results strongly argue for the importance of longitudinal studies when investigating early changes in synaptic physiology.

Prenatally treated VPA rats show behavioral deficits that share similarities with core autistic behaviors, in common with a small percentage of children that develop autism due to an exposure to VPA during pregnancy (Roullet et al., 2013). A validation of our observations would be to link autism associated behaviors in the VPA rat at specific ages with deficits in mPFC plasticity. Consistent with a delay in normalizing synaptic currents in early postnatal development, VPA rats and mice show a latency in nest-seeking behavior and bedding odor discrimination compared to control pups (Schneider and Przewłocki, 2005; Roullet et al., 2013), although these behaviors are not mPFC dependent. Deficits in social interaction are more clearly linked to abnormalities in mPFC function and these are consistently reported in the VPA rat (Roullet et al., 2013). However, from the earliest time points (pre-weaning, (Roullet et al., 2010)), into adolescence and adulthood social deficits are found in VPA exposed rats (Schneider and Przewłocki, 2005; Dufour-Rainfray et al., 2010; Kim et al., 2011). Therefore a direct link to social behavior and age dependent changes in NMDAR mediated LTP does not appear to be present. This of course does not exclude the importance of increased synaptic NMDAR and LTP in the formation of a locally hyper-connected state in the VPA rat pup (Rinaldi et al., 2008a,b). Other longitudinal tests of mPFC linked behaviors have not yet been reported in the VPA rat, although an adulthood deficit in radial maze learning is present (Narita et al., 2010). Ultimately a systematic test of mPFC dependent behaviors from adolescence to adulthood will be necessary to identify the specific late-onset behavioral deficits which our findings allude to.

## Conflict of interest statement

The authors declare that the research was conducted in the absence of any commercial or financial relationships that could be construed as a potential conflict of interest.

## References

[B1] Bromley R. L. Mawer G. Clayton-Smith J. Baker G. A. (2008). Autism spectrum disorders following in utero exposure to antiepileptic drugs. Neurology 71, 1923–1924 10.1212/01.wnl.0000339399.64213.1a 19047565

[B2] Chateauvieux S. Morceau F. Dicato M. Diederich M. (2010). Molecular and therapeutic potential and toxicity of valproic acid. J. Biomed. Biotechnol. 2010, 1–18 10.1155/2010/479364 20798865 PMC2926634

[B3] Christensen J. Grønborg T. K. Sørensen M. J. Schendel D. Parner E. T. Pedersen L. H. (2013). Prenatal valproate exposure and risk of autism spectrum disorders and childhood autism. JAMA 309, 1696–1703 10.1001/jama.2013.2270 23613074 PMC4511955

[B4] Cornish K. Turk J. Hagerman R. (2008). The fragile X continuum: new advances and perspectives. J. Intellect. Disabil. Res. 52, 469–482 10.1111/j.1365-2788.2008.01056.x 18444988

[B5] Dembrow N. C. Chitwood R. A. Johnston D. (2010). Projection-specific neuromodulation of medial prefrontal cortex neurons. J. Neurosci. 30, 16922–16937 10.1523/jneurosci.3644-10.2010 21159963 PMC3075873

[B6] Desai N. S. Casimiro T. M. Gruber S. M. Vanderklish P. W. (2006). Early postnatal plasticity in neocortex of Fmr1 knockout mice. J. Neurophysiol. 96, 1734–1745 10.1152/jn.00221.2006 16823030

[B7] Dufour-Rainfray D. Vourc’h P. Le Guisquet A.-M. Garreau L. Ternant D. Bodard S. (2010). Behavior and serotonergic disorders in rats exposed prenatally to valproate: a model for autism. Neurosci. Lett. 470, 55–59 10.1016/j.neulet.2009.12.054 20036713

[B8] Ebert D. H. Greenberg M. E. (2013). Activity-dependent neuronal signalling and autism spectrum disorder. Nature 493, 327–337 10.1038/nature11860 23325215 PMC3576027

[B9] Gandal M. J. Edgar J. C. Ehrlichman R. S. Mehta M. Roberts T. P. L. Siegel S. J. (2010). Validating *γ* oscillations and delayed auditory responses as translational biomarkers of autism. Biol. Psychiatry 68, 1100–1106 10.1016/j.biopsych.2010.09.031 21130222 PMC5070466

[B10] Geschwind D. H. Levitt P. (2007). Autism spectrum disorders: developmental disconnection syndromes. Curr. Opin. Neurobiol. 17, 103–111 10.1016/j.conb.2007.01.009 17275283

[B11] Goto Y. Yang C. R. Otani S. (2010). Functional and dysfunctional synaptic plasticity in prefrontal cortex: roles in psychiatric disorders. Biol. Psychiatry 67, 199–207 10.1016/j.biopsych.2009.08.026 19833323

[B12] Göttlicher M. Minucci S. Zhu P. Krämer O. H. Schimpf A. Giavara S. (2001). Valproic acid defines a novel class of HDAC inhibitors inducing differentiation of transformed cells. EMBO J. 20, 6969–6978 10.1093/emboj/20.24.6969 11742974 PMC125788

[B13] Iafrati J. Orejarena M. J. Lassalle O. Bouamrane L. Chavis P. (2013). Reelin, an extracellular matrix protein linked to early onset psychiatric diseases, drives postnatal development of the prefrontal cortex via GluN2B-NMDARs and the mTOR pathway. Mol. Psychiatry [Epub ahead of print]. 10.1038/mp.2013.148 23752244 PMC3965840

[B14] Jiang Y. Ehlers M. D. (2013). Modeling autism by SHANK gene mutations in mice. Neuron 78, 8–27 10.1016/j.neuron.2013.03.016 23583105 PMC3659167

[B15] Jung K.-M. Sepers M. Henstridge C. M. Lassalle O. Neuhofer D. Martin H. (2012). Uncoupling of the endocannabinoid signalling complex in a mouse model of fragile X syndrome. Nat. Commun. 3, 1080 10.1038/ncomms2045 23011134 PMC3657999

[B16] Kasanetz F. Manzoni O. J. (2009). Maturation of excitatory synaptic transmission of the rat nucleus accumbens from juvenile to adult. J. Neurophysiol. 101, 2516–2527 10.1152/jn.91039.2008 19244354

[B17] Kasanetz F. Lafourcade M. Deroche-Gamonet V. Revest J.-M. Berson N. Balado E. (2013). Prefrontal synaptic markers of cocaine addiction-like behavior in rats. Mol. Psychiatry 18, 729–737 10.1038/mp.2012.59 22584869

[B18] Kim K. C. Kim P. Go H. S. Choi C. S. Yang S.-I. Cheong J. H. (2011). The critical period of valproate exposure to induce autistic symptoms in Sprague–Dawley rats. Toxicol. Lett. 201, 137–142 10.1016/j.toxlet.2010.12.018 21195144

[B19] Kim K. C. Lee D.-K. Go H. S. Kim P. Choi C. S. Kim J.-W. (2013). Pax6-dependent cortical glutamatergic neuronal differentiation regulates autism-like behavior in prenatally valproic acid-exposed rat offspring. Mol. Neurobiol. [Epub ahead of print]. 10.1007/s12035-013-8535-2 24030726

[B20] Krueger D. D. Osterweil E. K. Chen S. P. Tye L. D. Bear M. F. (2011). Cognitive dysfunction and prefrontal synaptic abnormalities in a mouse model of fragile X syndrome. Proc. Natl. Acad. Sci. U S A 108, 2587–2592 10.1073/pnas.1013855108 21262808 PMC3038768

[B21] Lafourcade M. Elezgarai I. Mato S. Bakiri Y. Grandes P. Manzoni O. J. (2007). Molecular components and functions of the endocannabinoid system in mouse prefrontal cortex. PLoS One 2:e709 10.1371/journal.pone.0000709 17684555 PMC1933592

[B22] Lafourcade M. Larrieu T. Mato S. Duffaud A. Sepers M. Matias I. (2011). Nutritional omega-3 deficiency abolishes endocannabinoid-mediated neuronal functions. Nat. Neurosci. 14, 345–350 10.1038/nn.2736 21278728

[B23] Lee A. T. Gee S. M. Vogt D. Patel T. Rubenstein J. L. Sohal V. S. (2014). Pyramidal neurons in prefrontal cortex receive subtype-specific forms of excitation and inhibition. Neuron 81, 61–68 10.1016/j.neuron.2013.10.031 24361076 PMC3947199

[B24] Markram H. Rinaldi T. Markram K. (2007). The intense world syndrome–an alternative hypothesis for autism. Front. Neurosci. 15:6 10.3389/neuro.01.1.1.006.2007 18982120 PMC2518049

[B25] McElroy S. L. Keck P. E. Jr. Pope H. G. Jr. Hudson J. I. (1989). Valproate in psychiatric disorders: literature review and clinical guidelines. J. Clin. Psychiatry 50, 23–29 2494155

[B26] Meador K. J. Baker G. A. Browning N. Cohen M. J. Bromley R. L. Clayton-Smith J. (2013). Fetal antiepileptic drug exposure and cognitive outcomes at age 6 years (NEAD study): a prospective observational study. Lancet Neurol. 12, 244–252 10.1016/s1474-4422(12)70323-x 23352199 PMC3684942

[B27] Meador K. Reynolds M. W. Crean S. Fahrbach K. Probst C. (2008). Pregnancy outcomes in women with epilepsy: a systematic review and meta-analysis of published pregnancy registries and cohorts. Epilepsy Res. 81, 1–13 10.1016/j.eplepsyres.2008.04.022 18565732 PMC2660205

[B28] Mehta M. V. Gandal M. J. Siegel S. J. (2011). mGluR5-antagonist mediated reversal of elevated stereotyped, repetitive behaviors in the VPA model of autism. PLoS One 6:e26077 10.1371/journal.pone.0026077 22016815 PMC3189241

[B30] Meredith R. M. Holmgren C. D. Weidum M. Burnashev N. Mansvelder H. D. (2007). Increased threshold for spike-timing-dependent plasticity is caused by unreliable calcium signaling in mice lacking fragile X gene FMR1. Neuron 54, 627–638 10.1016/j.neuron.2007.04.028 17521574

[B31] Nadebaum C. Anderson V. Vajda F. Reutens D. Barton S. Wood A. (2011). The Australian brain and cognition and antiepileptic drugs study: IQ in school-aged children exposed to sodium valproate and polytherapy. J. Int. Neuropsychol. Soc. 17, 133–142 10.1017/s1355617710001359 21092354

[B32] Narita M. Oyabu A. Imura Y. Kamada N. Yokoyama T. Tano K. (2010). Nonexploratory movement and behavioral alterations in a thalidomide or valproic acid-induced autism model rat. Neurosci. Res. 66, 2–6 10.1016/j.neures.2009.09.001 19755133

[B33] Rasalam A. D. Hailey H. Williams J. H. G. Moore S. J. Turnpenny P. D. Lloyd D. J. (2005). Characteristics of fetal anticonvulsant syndrome associated autistic disorder. Dev. Med. Child Neurol. 47, 551–555 10.1017/s0012162205001076 16108456

[B34] Rinaldi T. Kulangara K. Antoniello K. Markram H. (2007). Elevated NMDA receptor levels and enhanced postsynaptic long-term potentiation induced by prenatal exposure to valproic acid. Proc. Natl. Acad. Sci. U S A 104, 13501–13506 10.1073/pnas.0704391104 17675408 PMC1948920

[B35] Rinaldi T. Perrodin C. Markram H. (2008a). Hyper-connectivity and hyper-plasticity in the medial prefrontal cortex in the valproic acid animal model of autism. Front. Neural Circuits 2:4 10.3389/neuro.04.004.2008 18989389 PMC2580056

[B36] Rinaldi T. Silberberg G. Markram H. (2008b). Hyperconnectivity of local neocortical microcircuitry induced by prenatal exposure to valproic acid. Cereb. Cortex 18, 763–770 10.1093/cercor/bhm117 17638926

[B37] Roullet F. I. Lai J. K. Y. Foster J. A. (2013). In utero exposure to valproic acid and autism—A current review of clinical and animal studies. Neurotoxicol. Teratol. 36, 47–56 10.1016/j.ntt.2013.01.004 23395807

[B38] Roullet F. I. Wollaston L. deCatanzaro D. Foster J. A. (2010). Behavioral and molecular changes in the mouse in response to prenatal exposure to the anti-epileptic drug valproic acid. Neuroscience 170, 514–522 10.1016/j.neuroscience.2010.06.069 20603192

[B39] Schneider T. Przewłocki R. (2005). Behavioral alterations in rats prenatally exposed to valproic acid: animal model of autism. Neuropsychopharmacology 30, 80–89 10.1038/sj.npp.1300518 15238991

[B40] Walcott E. C. Higgins E. A. Desai N. S. (2011). Synaptic and intrinsic balancing during postnatal development in rat pups exposed to valproic acid in utero. J. Neurosci. 31, 13097–13109 10.1523/jneurosci.1341-11.2011 21917793 PMC6623264

[B41] Watt A. J. van Rossum M. C. W. MacLeod K. M. Nelson S. B. Turrigiano G. G. (2000). Activity coregulates quantal AMPA and NMDA currents at neocortical synapses. Neuron 26, 659–670 10.1016/s0896-6273(00)81202-7 10896161

[B42] Zhao M.-G. Toyoda H. Ko S. W. Ding H.-K. Wu L.-J. Zhuo M. (2005). Deficits in trace fear memory and long-term potentiation in a mouse model for fragile X syndrome. J. Neurosci. 25, 7385–7392 10.1523/jneurosci.1520-05.2005 16093389 PMC6725289

